# Tumor-Associated Macrophages as Major Players in the Tumor Microenvironment

**DOI:** 10.3390/cancers6031670

**Published:** 2014-08-13

**Authors:** Theerawut Chanmee, Pawared Ontong, Kenjiro Konno, Naoki Itano

**Affiliations:** 1 Institute of Advanced Technology, Kyoto Sangyo University, Kita-ku, Kyoto 603-8555, Japan; E-Mail: k5372@cc.kyoto-su.ac.jp; 2 Division of Engineering (Biotechnology), Graduate School of Engineering, Kyoto Sangyo University, Kita-ku, Kyoto 603-8555, Japan; E-Mail: i1355034@cc.kyoto-su.ac.jp; 3 Department of Animal Medical Sciences, Faculty of Life Sciences, Kyoto Sangyo University, Kita-ku, Kyoto 603-8555, Japan; E-Mail: kkonno@cc.kyoto-su.ac.jp; 4 Department of Molecular Biosciences, Faculty of Life Sciences, Kyoto Sangyo University, Kita-ku, Kyoto 603-8555, Japan

**Keywords:** tumor-associated macrophage, M2 macrophage, tumor progression, angiogenesis, tumor metastasis, immunosuppression, cancer stem cells

## Abstract

During tumor progression, circulating monocytes and macrophages are actively recruited into tumors where they alter the tumor microenvironment to accelerate tumor progression. Macrophages shift their functional phenotypes in response to various microenvironmental signals generated from tumor and stromal cells. Based on their function, macrophages are divided broadly into two categories: classical M1 and alternative M2 macrophages. The M1 macrophage is involved in the inflammatory response, pathogen clearance, and antitumor immunity. In contrast, the M2 macrophage influences an anti-inflammatory response, wound healing, and pro-tumorigenic properties. Tumor-associated macrophages (TAMs) closely resemble the M2-polarized macrophages and are critical modulators of the tumor microenvironment. Clinicopathological studies have suggested that TAM accumulation in tumors correlates with a poor clinical outcome. Consistent with that evidence, experimental and animal studies have supported the notion that TAMs can provide a favorable microenvironment to promote tumor development and progression. In this review article, we present an overview of mechanisms responsible for TAM recruitment and highlight the roles of TAMs in the regulation of tumor angiogenesis, invasion, metastasis, immunosuppression, and chemotherapeutic resistance. Finally, we discuss TAM-targeting therapy as a promising novel strategy for an indirect cancer therapy.

## 1. Introduction

**Figure 1:**
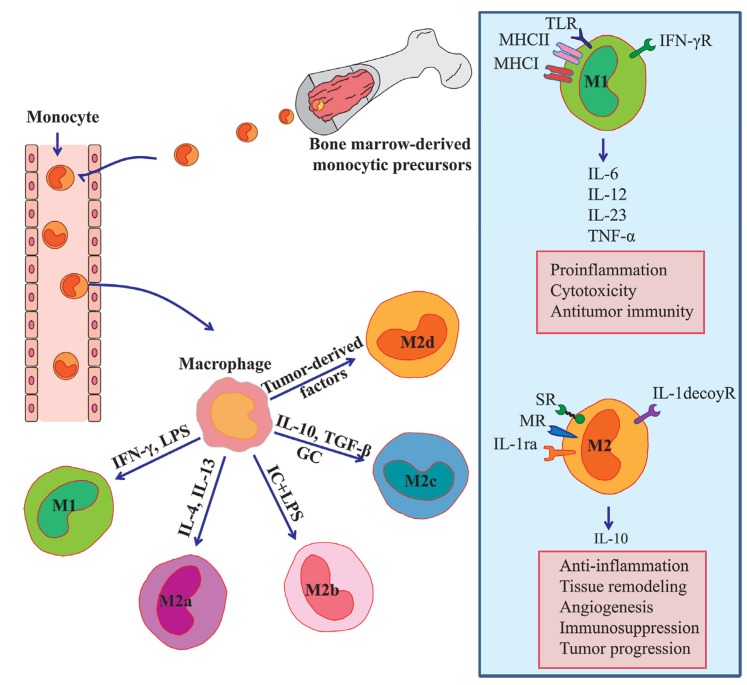
Macrophage polarization and its function. Tissue macrophages are derived from circulating monocyte and acquire either a classical M1 or alternative M2 phenotype depending on microenvironmental stimuli. M1 phenotype is driven by IFN-γ and LPS, and produce high levels of the pro-inflammatory cytokines such as IL-6, IL-12, IL-23, and TNF-α. M2 phenotype can be subdivided into M2a, M2b, M2c, and M2d according to different stimuli. M2 macrophages generally produced a high level of IL-10 and demonstrated with high levels of scavenger receptor, mannose receptor, IL-1 receptor antagonist, and IL-1 decoy receptor. M1 phenotype drives pro-inflammatory, cytotoxic and antitumor responses. In contrast, M2 phenotype promotes angiogenesis, immunosuppression, and tumor progression. LPS indicates lipopolysaccharide; IC, immune complex; GC, glucocorticoid; SR, scavenger receptor; MR, mannose receptor; IL-1ra, IL-1 receptor antagonist; TLR, Toll-like receptor; MHC, major histocompatibility complex.

Macrophages are innate immune cells that play a broad role in host defense and the maintenance of tissue homeostasis [1]. Tissue-resident and inflammatory macrophages originate from circulating bone marrow-derived monocytic precursors [2]. These precursor cells extravasate into target tissues where they differentiate into mature macrophages and polarize into diverse subsets that have different phenotypes in response to microenvironmental challenges [3]. Each polarized macrophage displays a differential expression profile of cytokines, enzymes, and cell-surface markers. In general, macrophages have been classified into two subsets: the classical M1 and the alternative M2 macrophages [4]. The M1 phenotype is driven by the Th1 cytokine interferon-*γ*, bacterial moieties such as lipopolysaccharide (LPS), and Toll-like receptor (TLR) agonists. They are characterized by the production of pro-inflammatory factors such as IL-6, IL-12, IL-23, and tumor necrosis factor-α (TNF-α). Furthermore, the M1 macrophages express high levels of the major histocompatibility complex class I and class II molecules that are required for the presentation of tumor-specific antigens. Thus, the M1 macrophages serve as a critical cellular component involved in the inflammatory response and antitumor immunity. Conversely, the M2 macrophages exert anti-inflammatory and pro-tumorigenic activities. The M2 macrophages can be further subdivided into subsets called M2a, M2b, M2c, and M2d. The Th2 cytokines such as IL-4 and IL-13 can stimulate the conversion of macrophage to M2a phenotype, whereas the activation of TLRs and immune complexes induces the M2b macrophages, and IL-10 polarizes the M2c subtype. Within the tumor, macrophages are a major stromal component, where they are commonly termed TAMs. Reports of recent research have demonstrated that TAMs exhibit functions similar to those of M2 macrophages and can be characterized as the M2d subtype [5,6] (Figure 1). During tumor development, tumor-infiltrating M1-polarized macrophages are generally characterized by an IL-12^high^ IL-10^low^ phenotype and promote immune responses that elicit tumor cell disruption. During late-stage tumor progression, TAMs generally switch to an M2-like phenotype characterized by an IL-12^low^ IL-10^high^ phenotype and low tumoricidal activity [5]. Such TAMs have been shown to provide a favorable microenvironment for tumor growth, tumor survival, and angiogenesis [7,8,9,10,11]. Although several studies have reported that TAMs exhibit an anti-inflammatory phenotype, in recent years, activated TAMs have been shown to produce multiple pro-inflammatory cytokines, such as IL-6 [12], that are involved in the induction of genes important to tumor cell cycle progression and apoptosis suppression [13]. Clinicopathological studies have indicated that patients with higher TAM densities have significantly worse relapse-free survival and overall survival rates [14,15]. Therefore, TAM infiltration appears to be a significant unfavorable prognostic factor for cancer patients, and may be a potentially useful prognostic marker of clinical outcomes.

## 2. Molecular Mechanisms of Monocyte/Macrophage Mobilization

**Figure 2:**
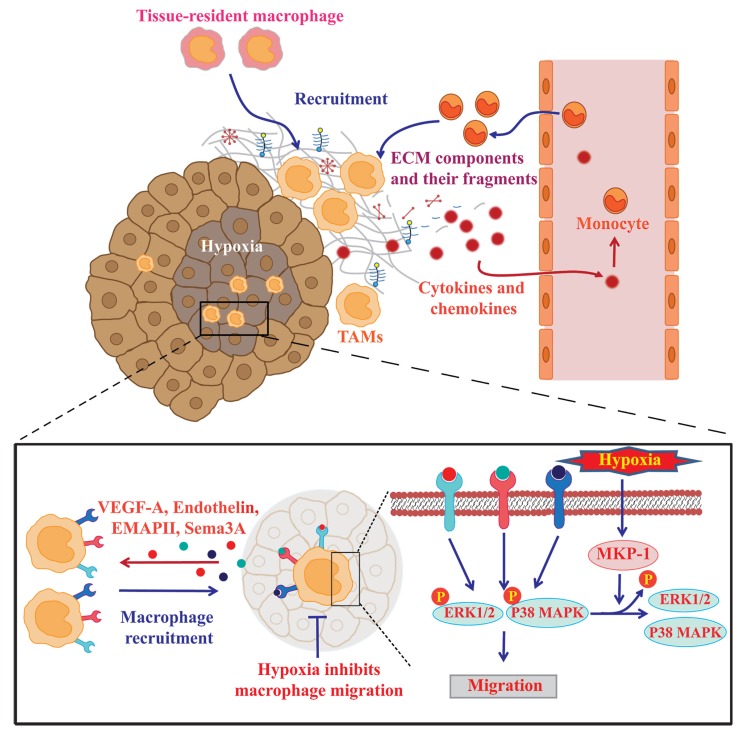
Mechanisms underlying the recruitment of monocytes/macrophages into tumors. Circulating monocytes and tissue-resident macrophages are mobilized into the tumor in response to multiple microenvironmental cues such as cytokines, chemokines, ECM components, and hypoxia. Hypoxic areas release higher amount of chemoattractants such as EMAPII, endothelin, and VEGF-A that enhance macrophage migration to these hypoxic sites. Hypoxia also restrains macrophages by decreasing their mobility through the upregulation of MKP-1 enzymes; this terminates the macrophage response to chemoattractants outside the hypoxic areas.

Circulating monocytes, derived from the bone marrow in the adult, give rise to tissue-resident and inflammatory macrophages throughout the body [3,16]. Similarly, TAMs are derived from circulating monocytes or tissue-resident macrophages [17]. Macrophage mobilization into tumor tissues is regulated by multiple microenvironmental cues such as cytokines, chemokines, extracellular matrix (ECM) components, and hypoxia (Figure 2).

### 2.1. Soluble Factors Mediating Monocyte/Macrophage Mobilization into Tumors

The recruitment of monocytes/macrophages into tumors is primarily regulated by cytokines, chemokines, and growth factors that are derived from tumor and stromal cells in the tumor microenvironment. Monocytes and macrophages migrate toward inflamed tissues under the influence of CCL2 (monocyte chemotactic protein-1; MCP-1) [18,19]. Clinicopathologically, the correlation between macrophage accumulation and the CCL2 level has been demonstrated in a broad spectrum of human tumors including breast [20], prostate [21], ovarian [22], and non-small lung cancers [23]. Experimental studies have also revealed the CCL2-dependent infiltration of macrophages into tumors [20]. For instance, systemic administration of CCL2-neutralizing antibodies in tumor-bearing mice or the short-hairpin RNA knockdown of CCR2 in the cancer cell lines significantly reduced tumor growth along with reduced TAM recruitment [21]. Tumor-derived CCL2 acts as a potent factor for Th2 polarization and shifts monocytes toward the M2-polarized macrophages [24,25]. Other CC chemokines including CCL3, CCL4, CCL5 (RANTES), and CCL22 (macrophage-derived chemokine) and some CXC chemokines, in particular CXCL8, may also be involved in TAM recruitment [6,26,27], because they are highly expressed in many human tumors and various tumor cell lines. Along with the chemokines, several cytokines such as colony-stimulating factor-1 (CSF-1) and endothelial monocyte-activating polypeptide II (EMAPII) have been implicated in the recruitment of monocytes into tumors [28,29]. Indeed, an elevated CSF-1 level correlates with marked macrophage infiltration in human metastatic breast cancer [30]. Xenograft experiments have further demonstrated that tumor cells transfected with *CSF-1* gene exhibited an increase in TAM infiltration [31]. Certain growth factors including vascular endothelial growth factor (VEGF), endothelin 2, and platelet-derived growth factor (PDGF) have also been reported to promote monocyte/macrophage recruitment [32,33,34]. Within the complex tumor microenvironment, the orchestrated actions of these soluble factors can synergistically accelerate the mobilization of monocytes/macrophages and the conversion of these cells to TAMs, which leads to further alterations in the tumor microenvironment.

### 2.2. Roles of ECM Components and Their Fragments in Controlling Macrophage Recruitment and Polarization

ECM serve as a structural scaffold for innate immune cell infiltration. Hyaluronic acid (HA), a major ECM component, has recently been implicated in monocyte/macrophage trafficking [35]. de la Motte and colleagues have shown that cable-like ECM structures were formed after extensive HA deposition on poly(I:C)-treated mucosal smooth muscle cells; these structures were involved in the adhesion and recruitment of monocytes through the association with HA receptor CD44 [36]. Several HA-binding partners, such as the inter-α-inhibitor (IαI) heavy chains, TNF-stimulated gene-6 (TSG-6), and HA-binding proteoglycan versican, are located along the strands of the cable-like structures and are expected to play crucial roles in ECM formation during an inflammatory reaction [37]. There was evidence that the HA cables produced by human intestinal smooth muscle cells in response to poly(I:C) treatment promoted leukocyte adhesion in a manner that was dependent on the presence of IαI heavy chains and TSG-6 [38]. After binding to high-molecular-weight HA, versican also cooperatively enhances leukocyte adhesion to these cables, suggesting that the pericellular HA complex provides a suitable scaffold for macrophage mobilization. Our recent study demonstrated the preferential engagement of immunosuppressive M2 macrophages in a HA- and versican-rich tumor microenvironment [35].

Certain ECM molecules and their proteolytic fragments have been shown to act as inflammatory stimuli for the recruitment of innate immune cells and the expression of pro-inflammatory genes [39]. Elastin fragments generated by macrophage-derived matrix metalloproteinase (MMP)-9/12 exhibit a monocyte chemotactic activity [40]. Denatured and fragmented collagen I also functions as a strong chemoattractant for macrophages. Alternatively, TAM-derived oncofetal fibronectin not only promotes cancer cell invasion but also stimulates monocyte migration [41]. Soluble biglycan and its fragments act on macrophages to produce both TNF-α and MIP-2 in a manner that is dependent on TLR2 and TLR4, and thereby play a positive role in macrophage recruitment and activation [42]. Tenascin-C induces cytokine synthesis in macrophages as an endogenous activator of TLR4 in arthritic joint disease [43]. Because elevated tenascin-C expression is often observed in the chronic inflammation of tumor stroma, an analogous mechanism may play an important role in inflammation in the tumor microenvironment. Kim and colleagues recently found that versican activated macrophages via TLR2 and its co-receptors TLR6 and CD14 [44]. Oligosaccharides generated by hyaluronidase-catalyzed digestion of high-molecular-weight HA also utilize both TLR2 and TLR4 to stimulate inflammatory gene expression in macrophages and act as an endogenous danger signal [45]. Tumor-derived HA fragments have also been shown to promote the development of immunosuppressive M2 macrophages by triggering a transient early activation of monocytes [46].

### 2.3. Hypoxia Promotes Macrophage Recruitment into Hypoxic Areas

Several pieces of evidence have shown that advanced solid tumors exhibit hypoxic areas within the tumor mass and that a high number of TAMs accumulate in the avascular areas of a wide range of human tumors [33]. VEGF-A [47], endothelin-2 [48], and EMAPII [49] have been implicated in the hypoxia-induced recruitment of macrophages. Deletion of myeloid-derived VEGF-A has been shown to reduce vascularization in solid tumors [50]. Casazza and colleagues demonstrated that TAM mobilization was dependent on the Semaphorin3A/Neuropilin-1 (Sema3A/Nrp1) signaling pathway. Because Sema3A acts as a chemoattractant for TAMs through binding to the Nrp1/PlexinA1 (pA1)/PlexinA4 (pA4) homoreceptor complex, hypoxia-dependent induction of Sema3A may promote TAM migration toward the hypoxic area [51].

Once TAMs reach the hypoxic areas, hypoxia directs the macrophages toward a pro-tumorigenic phenotype by altering the gene expression profiles. Hypoxia-inducible factor (HIF)-1α is a key transcription factor that regulates hypoxia-induced gene expression [52,53]. Hypoxic induction of CXC receptor 4 (CXCR4) in monocytes and macrophages is dependent on HIF-1α, which is paralleled by increased chemotactic responsiveness to its specific ligand CXCL12 [54]. HIF-1α also induces CXCL12 expression in direct proportion to the reduced oxygen tension at hypoxic sites [55]. As such, CXCL12 recruits CXCR4-expressing circulating monocytes/macrophages to the hypoxic areas within tumors. Several groups have reported that hypoxia upregulates VEGF-A expression in TAMs in a HIF-1α-dependent manner [56]. Increased expression of the macrophage chemoattractants further elicits TAMs into the hypoxic areas by generating a positive feedback loop.

On the other hand, hypoxia may also inhibit TAM migration and thereby cause them to accumulate within hypoxic areas. One possible mechanism is hypoxic induction of the enzyme mitogen-activated protein kinase phosphatase (MKP)-1 in TAMs. Evidence from a number of studies has suggested that MKP-1 terminates the response of TAMs to chemoattractants by inactivating ERK1/2 and p38 MAP kinase signaling pathways and thus keeps them at hypoxic sites [57]. Hypoxia also suppresses the expression of the chemokine receptors, CCR2 and CCR5, on macrophages and inhibits cell migration in response to the ligands [58].

## 3. Polarization of Macrophages toward the Pro-Angiogenic Phenotype

The tumor microenvironment often directs macrophage polarization from the M1 state that possesses inflammatory and antitumorigenic properties to the M2 state that has anti-inflammatory, pro-angiogenic, and pro-tumorigenic properties. TAMs generally have M2-like phenotypes that have the potential to secrete pro-angiogenic factors [59]. However, the specific phenotype of TAMs actually depends on the tumor progression stage [60]. In the early or regression stages of tumors, TAMs adopt the M1-like phenotype for the inhibition of angiogenesis in conjunction with the activation of tumor immunity. In contrast, TAMs shift to a M2-like state to enhance tumor angiogenesis in advanced tumors [60,61]. The polarization of macrophages toward the pro-angiogenic phenotype is regulated by hypoxia and the various signals derived from tumor and stromal cells [11,62]. Emerging evidence has suggested that the angiogenic switch in tumors depends on macrophage infiltration [63,64,65]. For instance, the pancreatic islet cancer model of CSF-1-deficient *op*/*op* mice displayed a substantial reduction in macrophage infiltration, angiogenic switching, and cumulative tumor burden [66]. Similarly, selective macrophage depletion using liposome-encapsulated clodronate (clodrolip) reduced angiogenesis in a transplanted tumor model [35].

TAMs secrete a wide range of pro-angiogenic mediators, including basic fibroblast growth factor, thymidine phosphorylase, urokinase-type plasminogen activator (uPA), and adrenomedullin (ADM), to facilitate tumor angiogenesis. Chen and colleagues found that infiltrating TAMs produced ADM when they interacted with melanoma cells. ADM also enhanced endothelial cell proliferation and tube formation by stimulating endothelial nitric oxide synthase [67], suggesting the pro-angiogenic action of TAM-derived ADM. TAMs sense hypoxia in avascular areas within tumors and release VEGF-A, a very potent pro-angiogenic factor [47,68]. Bingle and colleagues demonstrated that macrophage-derived VEGF-A in solid tumors contributed to the initiation of tumor angiogenesis with an increased number of vessels and branches. In the absence of macrophages, however, tumor angiogenesis was delayed [69]. At the hypoxic sites, HIF-1α upregulates VEGF-A expression in TAMs [70]. TAMs also secrete hypoxia-inducible proteolytic enzymes such as MMP-1 [71] and MMP-7 [72]. CD45^+^ monocytic cells, which are recruited into tumors during hypoxia facilitated tumor angiogenesis by MMP-9 secretion [73]. These MMPs releases sequestered VEGF from the ECM to accelerate tumor angiogenesis.

Tie2-expressing monocytes (TEMs) are a subset of circulating and tumor-infiltrating monocytes that express the angiopoietin receptor Tie2 and promote tumor angiogenesis. Various phenotypic differences have emerged among TEMs, Tie2-negative monocytes, and TAMs. Similar to TAMs, TEMs express high levels of the pro-angiogenic MMP-9 and VEGF-A when compared with Tie2-negative monocytes [10]. Angiopoietin-2, which is secreted from both tumor cells and vasculature, markedly induces an M2-like phenotype in TEMs by upregulating IL-10 and mannose receptor expression [10] while decreasing TNF-α and IL-12 expression [74]. Furthermore, TEMs reportedly exhibited greater angiogenic potential than TAMs upon coinoculation with tumor cells in a mouse model [75]. Despite expressing high levels of TLR4, TEMs exhibit reduced pro-inflammatory activity in response to LPS [76].

## 4. Macrophage-Dependent Promotion of Tumor Invasion and Metastasis

**Figure 3:**
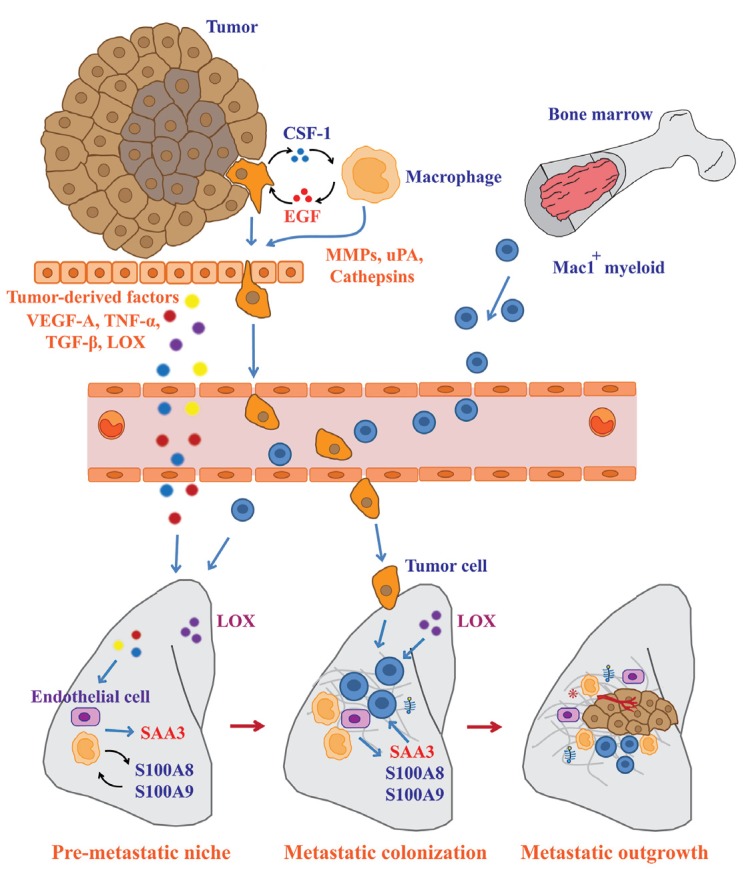
TAMs support the dissemination of tumor cells from primary to secondary sites. Metastatic processes begin with the invasion of tumor cells through the surrounding ECM, intravasation into the circulation, extravasation, and colonization at secondary sites. TAMs modulate the tumor microenvironment to allow tumor cell invasion by secreting growth factors and proteolytic enzymes. Remodeling of distant microenvironments can be induced by tumor-derived factors prior to the colonization of primary tumor cells. These factors stimulate local macrophages and endothelial cells to promote the recruitment of Mac1^+^ myeloid cells. Mac1^+^ myeloid cells are involved in increased ECM remodeling and pre-metastatic niche formation to allow metastatic tumor colonization. After the successful seeding of tumor cells at secondary sites, recruitment of macrophages and stromal cells are required to support the metastatic outgrowth.

TAMs are believed to directly and indirectly affect the metastatic process of tumor cells by modulating the tumor microenvironment [77,78]. Wyckoff and colleagues have developed a novel system to investigate the metastatic behavior of tumor cells using real-time imaging. Multi-photon imaging demonstrated that tumor cell intravasation occurs in association with TAMs in mammary tumors, which lends support to the notion that TAMs stimulate tumor metastasis [79]. Mechanistically, tumor cells synthesize CSF-1 in order to promote macrophage migration and macrophage-derived epidermal growth factor (EGF) enhances tumor cell invasion. Inhibition of CSF-1 or EGF abrogated the migration of both types of cells [80]. Thus, the paracrine loop between these factors has been proposed for the control of tumor cell invasion (Figure 3).

Intrinsic and extrinsic signals originating from tumor cells and the tumor microenvironment actively support the metastatic processes of the tumor cells. Matrix components in the tumor microenvironment have been reported to modulate the function of TAMs to enhance metastatic survival and the outgrowth of tumor cells. Versican, a large chondroitin sulfate proteoglycan, which is more frequently expressed in malignant tumors and is implicated in tumor progression, has been shown to activate macrophages. Kim and colleagues demonstrated that Lewis lung carcinoma cells produced a large amount of versican that activated macrophages via TLR-2/TLR-6 and enhanced metastatic tumor growth [44]. Macrophages are the major source of various proteolytic enzymes such as cathepsins, MMPs, and serine proteases. It has been believed that macrophage-derived proteases degrade the surrounding ECM and thereby allow cancer cells to invade this barrier (Figure 3). Gocheva and colleagues found that the depletion of TAM-derived cathepsin B and S impaired tumor invasion [81], signifying the important role of TAM-derived proteases in tumor invasion. Similarly, TAMs significantly increased the migration and invasion of cancer cells by highly expressing uPA [82].

## 5. Significant Contribution of Macrophages to Pre-metastatic Niche Formation

Cancer metastasis is a multistep process that requires the concerted action of both primary tumors and distant organs. Before metastatic tumor cells colonize, secondary organs undergo early changes in their local microenvironment, termed the “pre-metastatic niche.” Recent studies have shown that macrophage recruitment is an important step for metastatic cell survival and pre-metastatic niche formation. Gil-Bernabe and colleagues found that tumor-derived tissue factor (coagulation factor III or CD142) stimulated clot formation and enhanced subsequent tumor cell survival at the metastatic site by recruiting CD11b^+^/CD68^+^/F4/80^+^/CX3CR1^+^ macrophages [83]. Erler and colleagues identified the role of lysyl oxidase (LOX) in the formation of the pre-metastatic niche (Figure 3). LOX, a copper-dependent amine oxidase, stabilizes the ECM network by cross-linking collagen and elastin. LOX secreted from tumor cells forms the cross-links of collagen IV in the basement membranes at the pre-metastatic sites. CD11b^+^ myeloid cells then adhere to the cross-linked collagen IV and produce MMP-2. The collagen IV peptide cleaved by MMP-2 then enhances the further recruitment of CD11b^+^ cells as a chemoattractant [84]. This positive feed-forward loop eventually increases the extracellular matrix remodeling and the creation of the pre-metastatic niche.

Hiratsuka and colleagues have demonstrated that primary tumors stimulated the expression of S100A8 and S100A9 proteins in the lung by secreting VEGF-A, TNF-α, and TGF-β. Both S100A8 and S100A9 proteins subsequently induced the recruitment of macrophage antigen 1 (Mac1)-positive myeloid cells to the pre-metastatic milieu. Neutralization of these factors with specific antibodies blocked the infiltration of Mac1^+^ myeloid cells and the migration of cancer cells from primary tumors to the lung [85], suggesting that the S100A8 and S100A9 proteins could play a critical role in the formation of the pre-metastatic niche. They also found that S100A8 and S100A9 induced the expression of serum amyloid A3 in alveolar macrophages as well as in endothelial cells [86]. Serum amyloid A3 and S100A8 increased vascular permeability through the engagement of TLR4 and MD-2, a co-receptor for TLR4, on lung endothelial cells [87]. Because tumor cells preferentially metastasize to the hyperpermeable region of the vasculature in the target organs [88], their findings propose a novel mechanism for the macrophage-dependent enhancement of metastatic processes (Figure 3).

## 6. TAM-Mediated Immunosuppression in Tumor Microenvironment

**Figure 4:**
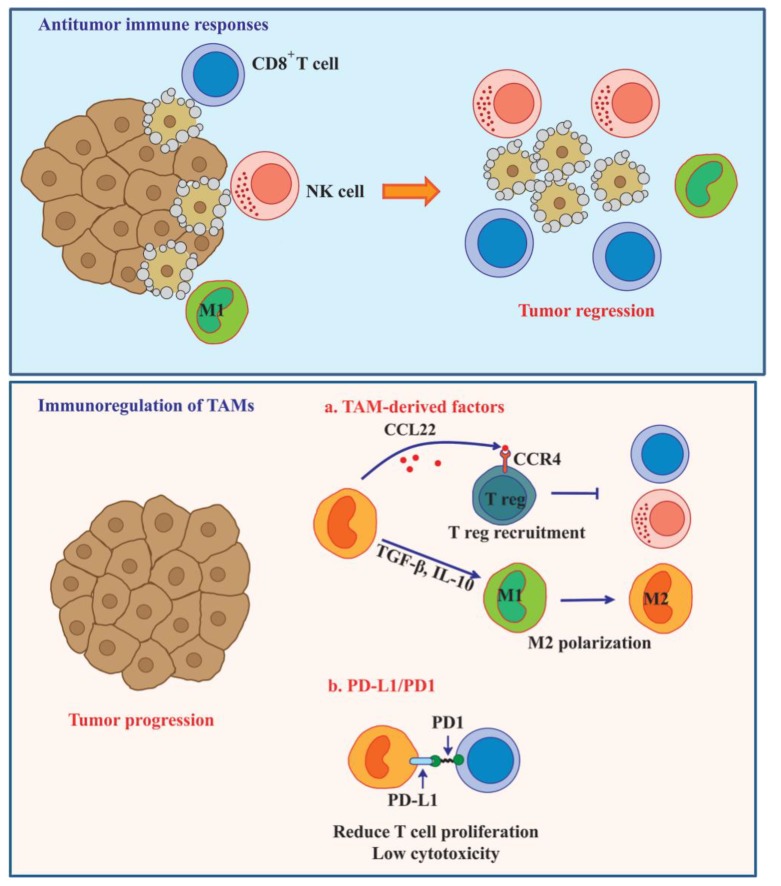
TAMs induce immune dysfunction and enhance tumor progression. Immune system promotes the elimination of tumor by the function of CD8^+^ T cell, NK cell, and M1 macrophage. These immune responses are modulated or suppressed by the tumor microenvironment to allow tumor cells survival. The inefficacy of immune cells to destroy tumor is regulated by TAMs. TAMs support the immunosuppression in tumor by secreting several factors such as CCL22, IL-10, and TGF-β. Treg recruitment into tumor is controlled by the CCL22/CCR4 axis. These cells suppress immune surveillance through multiple mechanisms including inhibition of T cell proliferation and activation or inhibition of NK cell cytotoxicity. Releasing of immunosuppressive factors such as IL-10 and TGF-β can also polarize M1 to M2 macrophage. TAMs also directly inhibit T cell proliferation and cytotoxicity by the PD-L1/PD1 signaling axis.

Escape of tumor cells from immunosurveillance is one of critical events that regulate tumor growth, survival, and metastasis. TAMs in a M2 macrophage-like state possess a poor antigen-presenting capability and suppress the immune response of T cells by releasing the immunosuppressive factors, IL-10 and TGF-β [89]. Kuang and colleagues observed that TNF-α and IL-10 secretion from activated monocytes strongly induced the expression of programmed cell death 1 (PD-L1) in an autocrine manner. PD-L1-positive monocytes induced T cell dysfunction, as defined by the presence of low cytotoxicity to tumor cells and a reduction in T cell proliferation [90]. Blocking PD-L1 with a specific antibody improved specific T cell immunity, suggesting that PD-L1 limits the capacity of T cells to eliminate tumor cells (Figure 4).

An increased number of regulatory T cells are commonly found in various tumors [91,92,93,94]. CD4^+^CD25^+^FOXP3^+^ tumor-infiltrating regulatory T cells (Treg) counteract T cell-mediated immune responses. Intratumoral Treg trafficking is mediated through the CCL22/CCR4 axis [95]. In human ovarian carcinoma, TAMs produce the chemokine CCL22, which is one of the CCR4 ligands, as a mediator for the trafficking of Tregs to the tumor [93,96] (Figure 4).

## 7. Roles of TAMs in Self-Renewal and Chemotherapeutic Resistance of Cancer Stem Cells

Cancer stem cells (CSCs) represent a population of cancer cells that possess the ability to self-renew and give rise to malignant progeny. CSCs have also emerged as a major driving force governing tumor recurrence due to their resistance to chemotherapeutic agents [97,98]. TAMs have been reported to control both the self-renewal and drug resistance of CSCs through a complex network of cytokines, chemokines, growth factors, and extracellular matrix molecules. Yi and colleagues found that glioma-initiating cells produced CCL2, CCL5, VEGF-A, and neurotensin at higher levels than the glioma cells; these findings suggest that CSCs play an important role in TAM recruitment by secreting macrophage chemoattractants [99]. Another study also proposed various roles for TAMs in CSC self-renewal through the paracrine loop of the EGF signaling pathway. Yang and colleagues reported that TAMs promoted CSC-like phenotypes in murine breast cancer cells by activating the EGF signaling pathway. The EGF signal stimulated Stat3 phosphorylation and induced Sox-2 expression that was required for the maintenance of CSC phenotypes [100]. Matrix components in tumor microenvironment can also modulate the function of TAMs to support CSC self-renewal. Okuda and colleagues reported that HA produced by metastatic breast CSCs enhanced tumor invasion and metastasis into the bone microenvironment [101]. Mechanistically, HA promotes the interaction between TAMs and CSCs followed by PDGF-BB secretion from TAMs. The TAM-derived PDGF-BB then activates fibroblasts and osteoblasts to support CSC self-renewal through the induction of fibroblast growth factor (FGF)-7 and FGF-9 expression.

## 8. Future Prospects in TAM-Targeting Cancer Therapy

Increasing evidence has demonstrated that TAM accumulation is associated with poor clinical prognosis and resistance to cancer therapy [102,103]. This is in part from the immunosuppressive and tumor-promoting activities of TAMs. Based on these findings, TAMs are emerging as attractive targets for therapeutic intervention. Recently, several studies on TAM-targeting cancer therapy have focused on the following strategies: inhibition of macrophage recruitment, conversion of pro-tumorigenic M2 to the antitumor M1 phenotype, and suppression of TAM survival.

As described above, tumor- and stroma-derived chemoattractants facilitate macrophage recruitment into tumors. Thus, the inhibition of macrophage recruitment through the modulation of the chemoattractants may become a more effective cancer therapy. Indeed, the pharmacological inhibition of CCL2 with Bindarit reduced macrophage recruitment and suppressed tumor growth [104]. The selective inhibition of VEGFR2 with a specific monoclonal antibody reduced macrophage infiltration and tumor growth [105]. Colony-stimulating factor 1 receptor (CSF-1R)-targeted therapy may be another strategy for manipulating intratumoral macrophage numbers. The humanized monoclonal antibody RG7155 potently inhibits CSF-1R dimerization. The clinical activity of RG7155 was evaluated in patients with diffuse-type giant cell tumor and was shown to induce a striking reduction in the CSF-1R^+^CD163^+^ macrophage population within tumor tissues [106]. PLX3397, a potent tyrosine kinase inhibitor of CSF-1R, enhanced the efficacy of immunotherapy by decreasing macrophage infiltration and activating tumor-infiltrating lymphocytes [107]. In line with these strategies, other macrophage chemoattractants such as CCL5 and CXCL12 may be promising targets for drug development [108,109,110].

Because the classical M1 macrophage possesses antitumor activity, the polarization from tumor-promoting M2 to tumoricidal M1 macrophages appears to be a better potential target for cancer therapy. Several studies have reported that TLR activation leads tumor-supporting macrophages to tumoricidal effectors. In tumor-bearing mice, Shime and colleagues found that activation of TLR3/Toll-IL-1 receptor domain-containing adaptor molecule 1 by Poly(I:C) rapidly induced the production of pro-inflammatory cytokines and thereafter accelerated M1 macrophage polarization [111]. Zoledronic acid, a clinical drug for cancer therapy, has been found to inhibit spontaneous mammary carcinogenesis by reverting macrophages from the M2 phenotype to the M1 phenotype [112]. Emerging evidence has indicated that intratumoral blood vessels contain structural and functional abnormalities. This abnormal tumor vasculature alters the tumor microenvironment and influences tumor progression and responses to cancer therapy. Thus, the restoration of normal blood vessel structure and function may enhance cancer treatment. The re-education of TAMs within the tumor could block the pro-tumorigenic effects of TAMs through vascular normalization. Zhang *et al*. reported that polarization from an M2 to M1 phenotype suppressed mammary tumor growth and angiogenesis *in vivo* [113]. Recently, Rolny *et al.* demonstrated that histidine-rich glycoprotein (HRG) inhibited tumor growth and metastasis by inducing macrophage polarization and vessel normalization via downregulation of the placental growth factor (PlGF) [114]. 

It has been generally believed that suppression of TAM survival improves the therapeutic approach to tumors. One of the effective strategies to kill TAMs is to directly induce apoptosis in TAMs using chemical or synthetic drugs. Trabectedin (ET-743) is an antitumor drug for the treatment of soft tissue sarcoma and relapsed platinum-sensitive ovarian cancer patients. This compound can selectively deplete TAMs in tumor patients by activating the extrinsic apoptotic pathway via TRAIL receptors [115]. Since Trabectedin not only targets TAM function but also directly affects monocyte/macrophage-mediated host defense [115], developing TAM-specific agents would be therefore required to limit the side effects. Cieslewicz and colleagues developed a unique peptide M2pep that preferentially binds to M2 macrophages. A study in tumor-bearing mice demonstrated that M2pep carrying a pro-apoptotic peptide selectively killed TAMs and improved survival rates in the mice [116].

## 9. Conclusions 

TAMs comprise a part of the tumor microenvironment in aggressive tumors, and their mobilization into tumor tissues is a critical event in malignant progression. Macrophage recruitment and polarization are regulated by multiple cues from tumor cells and the tumor microenvironment. Upon direct or indirect interaction with TAMs and tumor microenvironments, TAMs synthesized and released a vast diversity of growth factors, cytokines, chemokines, ECM components, and protease enzymes. These TAM-derived factors then promoted matrix remodeling, angiogenesis, anti-immune responses, and tumor progression. TAMs are also involved in the resistance of CSCs to chemotherapeutic drugs. Because high TAM infiltration is associated with poor prognosis and therapeutic failure in cancer patients, reprogramming of TAM toward an antitumor M1 phenotype, inhibition of TAM recruitment, and suppression of TAM survival may become the foci of promising novel cancer therapies.
